# Metabolomic signature between diabetic and non-diabetic obese patients: A protocol for systematic review

**DOI:** 10.1371/journal.pone.0296749

**Published:** 2024-01-17

**Authors:** Yuxing Tai, Xiaoqian Yang, Xiaochao Gang, Zhengri Cong, Sixian Wang, Peizhe Li, Mingjun Liu

**Affiliations:** 1 Department of Acupuncture and Tuina, Changchun University of Chinese Medicine, Changchun, Jilin, China; 2 Jilin Ginseng Academy, Changchun University of Chinese Medicine, Changchun, Jilin, China; 3 Acupuncture and Massage Center of the Third Afliated Clinical Hospital, Changchun University of Chinese Medicine, Changchun, Jilin, China; The University of Mississippi Medical Center, UNITED STATES

## Abstract

**Background:**

Type 2 diabetes mellitus (T2DM) is a chronic and progressive condition defined by hyperglycemia caused by abnormalities in insulin production, insulin receptor sensitivity, or both. Several studies have revealed that higher body mass index (BMI) is associated with increasing risk of developing diabetes. In this study, we perform a protocol for systematic review to explore metabolite biomarkers that could be used to identify T2DM in obese subjects.

**Methods:**

The protocol of this review was registered in PROSPERO (CRD42023405518). Three databases, EMBASE, PubMed, and Web of Science were selected to collect potential literature from their inceptions to July December 2023. Data for collection will include title, authors, study subjects, publication date, sample size, detection and analytical platforms, participant characteristics, biological samples, confounding factors, methods of statistical analysis, the frequency and directions of changes in potential metabolic biomarkers, and major findings. Pathway analysis of differential metabolites will be performed with MetaboAnalyst 5.0 based on the Kyoto Encyclopedia of Genes and Genomes (KEGG) and the Human Metabolome Database.

**Results:**

The results of this systematic review will be published in a peer-reviewed journal.

**Conclusion:**

This systematic review will summarize the potential biomarkers and metabolic pathways to provide a new reference for the prevention and treatment of T2DM in obese subjects.

## Introduction

Type 2 diabetes mellitus (T2DM) is one of the most significant global health problems without a definitive cure. The prevalence of T2DM is projected to affect 366 million adults by 2030, showing a significant increase [1]. This rate of increase was evident, accompanied by rapid economic growth that coincided with a rise in diabetes and its clinical complications. Due to its long-term consequences, T2DM is now one of the leading causes of death in the world [2]. Despite continuous advancements in diabetes care and the development of new therapies, a substantial proportion of T2DM patients fail to achieve their desired glycemic targets. Less than 30% of individuals with T2DM reach glycated hemoglobin (HbA1c) levels below 7.0% [3, 4]. These statistics underscore the need for early intervention and preventive strategies based on diagnostic markers to improve glycemic control. Moreover, intensive glucose therapy in the early period has been shown to reduce the long-term risk of microvascular complications and mortality from any cause [5]. Consequently, early and accurate diagnosis of diabetes has become an important goal in current clinical practice.

Obesity is considered a chronic metabolic disease that increases the risk of T2DM, which is preceded by insulin resistance (IR). The increasing incidence rate of obesity worldwide in the past century has further intensified the prevalence of diabetes [6]. In this context, weight management is strongly promoted by current standards for the management of T2DM, because even moderate weight loss (5%) improves insulin sensitivity and other comorbidities related to obesity [7–9]. Although obesity is a predominant trigger and prodrome for diabetes, not all obese individuals develop glucose metabolic disorders [10]. The transition from obesity to clinically diagnosed diabetes may take a long subclinical period, which suggests that the predictive value of body weight or body mass index (BMI) for metabolic disorders is limited. Therefore, it is important to identify novel biomarkers for better stratification and appropriate treatment of high-risk subjects.

Over the past few years, inspired by the necessity of preventing the diabetes epidemic, researchers have made significant discoveries using metabolomics technologies to gain a deeper understanding of obesity-induced T2DM [11–13]. Metabolomics, a high-throughput technique for biomarker discovery, can profile an individual’s metabolic state by analyzing small molecule metabolites (<1500 Da) in the internal environment [14, 15]. The number of metabolites in humans ranges from 2,000 to 20,000 [16], and two primary analytical methods are primarily utilized for the quantification of metabolites (located in cells, tissues, or body fluids): nuclear magnetic resonance (NMR) and mass spectrometry (MS) [5–7], through a targeted or untargeted process [17]. Targeted analysis is a more quantitative approach, but it inevitably risks missing unknown or unexpected biomarkers. In contrast, non-targeted analysis can survey a wide range of metabolites in order to gain a deeper understanding of phenotypes and identify potential biomarkers [18]. The alterations in various metabolites have been reported to be associated with disorders in glycolipid metabolism [19]. Accordingly, non-targeted metabolomics can be used to predict the progression of T2DM, as well as future insulin resistance and impaired glucose tolerance, through the analysis of individuals with advanced obesity or metabolic syndrome [20, 21]. In this paper, we perform a systematic review protocol to investigate the differences in metabolomic profiling between diabetic and non-diabetic obese patients. The findings will help advance the personalized care of patients with T2DM and contribute to the development of new diagnostic and therapeutic options.

## Methods

The protocol for this review has been registered in PROSPERO (ID: CRD42023405518). Additionally, it will be reported following the guidelines of the Preferred Reporting Items for Systematic Review and Meta-Analysis (PRISMA) 2020 Statement [22]. Ethical approval is not required as this systematic review will retrieve scientific literature available in public databases.

### Inclusion criteria

All cross-sectional and longitudinal studies that use non-targeted metabolomics techniques to identify differential metabolites between individuals with diabetic obesity and those with non-diabetic obesity will be included. The literature included is limited to English language. However, animal studies, cellular studies, case reports, environmental studies, reviews, commentaries, and other studies that are repeatedly published will be excluded. Adult patients diagnosed with obesity and T2DM based on any recognized diagnostic criteria will be included, regardless of gender, race, and region.

### Search methods

We will search 3 public databases (Web of Science, Embase, PubMed) to collect relevant studies from their inception until July 2023. The following search terms will be used: metabolomics, T2DM, and obesity. The details of the search strategy are shown in Table 1.

**Table 1:** Search strategy.

Search #	Search terms
#1	metabolomics [Title/Abstract]
#2	metabonomics [Title/Abstract]
#3	metabolome [Title/Abstract]
#4	metabolic profiling [Title/Abstract]
#5	#1 OR #2 OR #3 OR #4
#6	diabetes [Title/Abstract]
#7	insulin resistance [Title/Abstract]
#8	T2DM [Title/Abstract]
#9	T2D [Title/Abstract]
#10	DM [Title/Abstract]
#11	#6 OR #7 OR #8 OR #9 OR #10
#12	body mass index [Title/Abstract]
#13	overweight [Title/Abstract]
#14	obesity [Title/Abstract]
#15	adiposity [Title/Abstract]
#16	obese [Title/Abstract]
#17	#12 OR #13 OR #14 OR #15 OR #16
#18	#5 AND #11 AND #17

T2DM = type 2 diabetes mellitus, T2D = type 2 diabetes, DM = diabetes mellitus.

### Study selection

**Fig 1:**
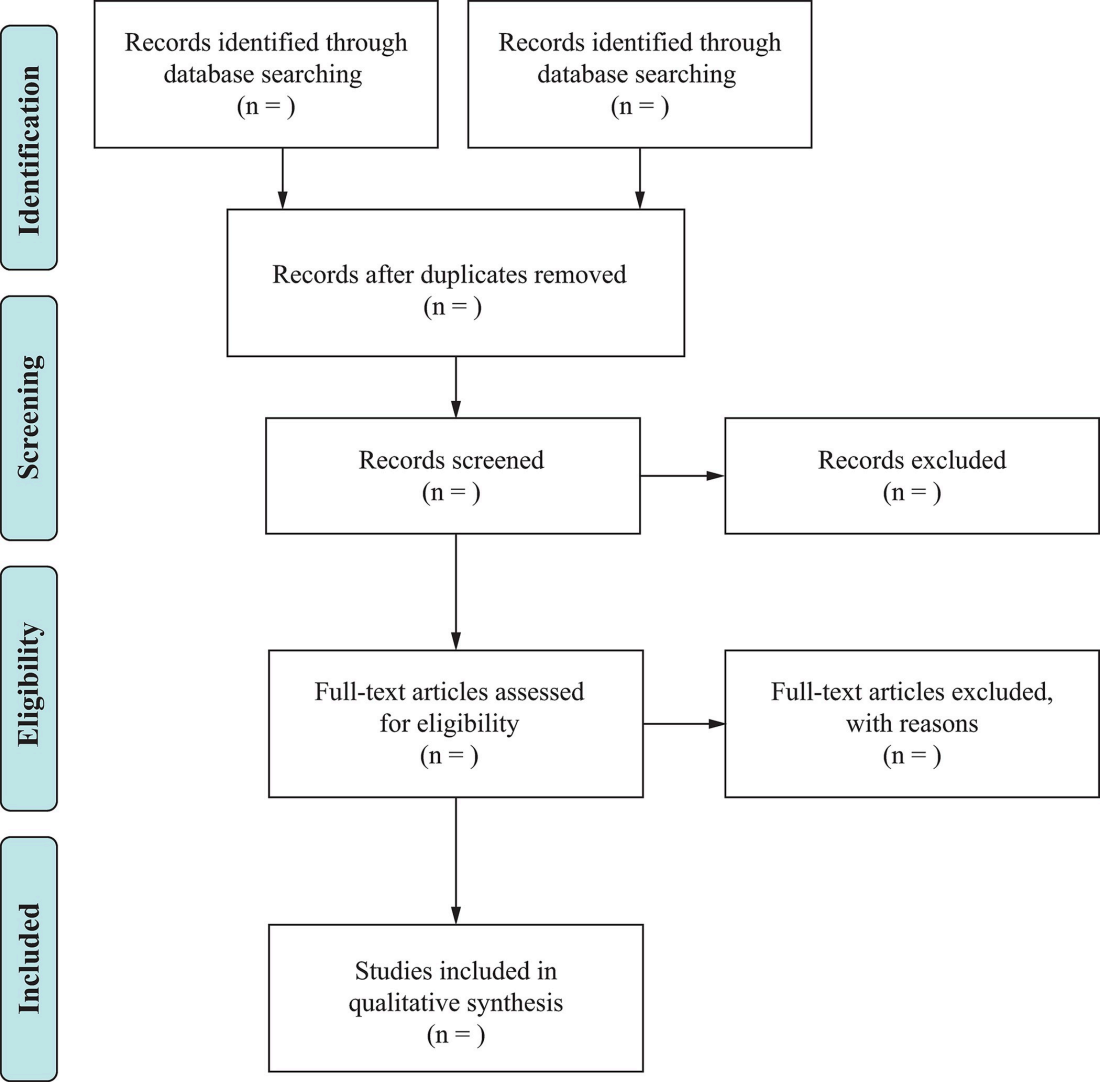
Flow chart of study.

Before conducting the search for potential studies, researchers will import the retrieved studies into EndnoteX7 (version 20), a document management software, to remove any duplicates. Two reviewers will then independently conduct a preliminary screening by reading the unique titles and abstracts to exclude any unqualified studies. The final included literature will be determined by the other two reviewers, who will assess the full text to gain a better understanding of the research details. Any conflicts concerning eligibility will be resolved by a senior author. The detailed process of study selection is summarized in a PRISMA flow chart (Fig 1).

### Quality assessment

We will assess the quality of the included studies based on the guide of QUADOMICS [23], which is an adaptation of the Quality Assessment of Diagnostic Accuracy Assessment (QUADAS) for evaluating ‘-omics’-based research including in systematic reviews. The quality of evidence will be divided into high quality (QUADOMICS scores 9–16) and low quality (QUADOMICS scores 0–8) based on an equal distribution.

### Data extraction and pathway analysis

Data for collection includes title, authors, study subjects, publication date, sample size, detection and analytical platforms, participant characteristics, biological samples, confounding factors, methods of statistical analysis, the frequency and directions of changes in potential metabolic biomarkers, and major findings. Any disagreements regarding data extraction will be resolved through discussions or arbitrations with a senior researcher. To further investigate the underlying molecular mechanism, pathway analysis of differential metabolites will be performed using MetaboAnalyst 5.0, based on the Kyoto Encyclopedia of Genes and Genomes (KEGG) and Human Metabolome Database.

## Expected results

This systematic review will provide a comprehensive summary of metabolic profiling in diabetic and non-diabetic obese patients, and identify potential biomarkers with predictive value to predict T2DM.

### Discussion

With the advancement of high-throughput technology, metabolomics data sources have become increasingly abundant. To date, more than 4,000 metabolites have been confirmed in human samples using various metabolomics platforms and technologies [24]. The changes in metabolic profiles can indicate pathological mechanisms and provide potential biomarkers for the diagnosis and evaluation of several diseases [17]. Numerous studies have systematically evaluated the metabolic characteristics of osteoarthritis, obesity (including adults and children), metabolic syndrome, T2DM, and pre-diabetes, screening out new metabolic markers with significant diagnostic and therapeutic implications in the future [20, 25–27]. Unfortunately, no researcher has compared the differential metabolites between diabetic and non-diabetic obese patients in this way.

Insight into the molecular mechanisms underlying the development of type 2 diabetes from obesity remains limited. BMI continues to be widely used as a standard for grading obese individuals due to its strong association with adverse health outcomes, including T2DM. Notably, as demonstrated in many individuals, excessive fat accumulation does not directly lead to the occurrence of hyperglycemia, and a single biomarker lacks stability and specificity for diagnosing a disease [20]. The construction of the metabolite profile can enable the integration of multiple biomarkers, which may predict and prevent disease progression to pre-diabetes or diabetes. This is the first systematic review to uncove the metabolic changes that underlie the progression from obesity to diabetes.

## Supporting information

S1 Checklist PRISMA-P 2015 checklist. (DOCX)

## Abbreviations

T2DM: type 2 diabetes mellitus
PRISMA: preferred reporting items for systematic review and meta-analysis
KEGG: Kyoto encyclopedia of genes and genomes
